# Multi-analyser detector (MAD) for high-resolution and high-energy powder X-ray diffraction

**DOI:** 10.1107/S1600577520013223

**Published:** 2021-01-01

**Authors:** Alexander Schökel, Martin Etter, Andreas Berghäuser, Alexander Horst, Dirk Lindackers, Thomas A. Whittle, Siegbert Schmid, Matias Acosta, Michael Knapp, Helmut Ehrenberg, Manuel Hinterstein

**Affiliations:** a Institute for Applied Materials, Karlsruhe Institute of Technology (KIT), PO Box 3640, 76021 Karlsruhe, Germany; b Deutsches Elektronen-Synchrotron (DESY), Notkestrasse 85, 22607 Hamburg, Germany; c Helmholtz-Zentrum Dresden Rossendorf, FWKX@XFEL, Holzkoppel 4, 22869 Schenefeld, Germany; d Research Technology, IFW Dresden, PO Box 27 10 16, 01171 Dresden, Germany; e School of Chemistry, The University of Sydney, Sydney, NSW 2006, Australia; f Institute of Materials Science, Technische Universität Darmstadt, Alarich-Weiss-Straße 2, 64287 Darmstadt, Germany

**Keywords:** powder X-ray diffraction, high-resolution detectors, multi-analyser detectors, high-energy diffraction, strontium niobate titanate, strontium niobate zirconate, BZT–*x*BCT

## Abstract

For high-resolution powder diffraction in material science, high photon energies are necessary, especially for *in situ* and *in operando* experiments. For this purpose, a multi-analyser detector (MAD) was developed for the high-energy beamline P02.1 at PETRA III of the Deutsches Elektronen-Synchrotron (DESY). In order to be able to adjust the detector for the high photon energies of 60 keV, an individually adjustable analyser–crystal setup was designed. The adjustment is performed via piezo stepper motors for each of the ten channels. The detector shows a low and flat background as well as a high signal-to-noise ratio. A range of standard materials were measured for characterizing the performance. Two exemplary experiments were performed to demonstrate the potential for sophisticated structural analysis with the MAD: (i) the structure of a complex material based on strontium niobate titanate and strontium niobate zirconate was determined and (ii) an *in situ* stroboscopy experiment with an applied electric field on a highly absorbing piezoceramic was performed. These experiments demonstrate the capabilities of the new MAD, which advances the frontiers of the structural characterization of materials.

## Abstract 2

The layout and performance of a multi-analyser detector for high-resolution powder diffraction at an X-ray energy of 60 keV are described.

## 1. Introduction   

Over recent decades the powder-diffraction technique has developed into one of the most powerful and versatile techniques for structural characterization of materials. It is routinely used in laboratory setups and also in synchrotron and neutron facilities, especially for *in situ* and *in operando* experiments (Ehrenberg *et al.*, 2013 ▸, 2019 ▸). During this development, sophisticated experiments progressively demanded the continuous increase of photon energy (Ehrenberg *et al.*, 2013 ▸). Conventional powder-diffraction beamlines usually operate at rather soft photon energies of up to 20–30 keV owing to the low critical energies of the source (Knapp *et al.*, 2004 ▸; Patterson *et al.*, 2005 ▸; Wallwork *et al.*, 2007 ▸; Thompson *et al.*, 2009 ▸; Yang *et al.*, 2015 ▸; Lausi *et al.*, 2015 ▸). For some specific experiments, such as resonant scattering, even energies below 8 keV were used (Staub *et al.*, 2000 ▸, 2001 ▸; Ehrenberg *et al.*, 2000 ▸). For several applications in materials science, such as *in situ* or *in operando* studies, energies well above 30 keV are required to be able either to penetrate complete devices or to measure bulk materials with high absorption. A range of beamlines focus on this energy range (Fitch, 2004 ▸; Wang *et al.*, 2008 ▸; Fauth *et al.*, 2013 ▸; Dippel *et al.*, 2015 ▸). For total scattering applications, energies between 50 and 120 keV are typically employed in order to be able to access a large *Q* range (where *Q* is the scattering vector) (Shi *et al.*, 2013 ▸; Dippel *et al.*, 2015 ▸; Sutter *et al.*, 2016 ▸; Ren & Zuo, 2018 ▸; Billinge, 2019 ▸; Vaughan *et al.*, 2020 ▸).

In parallel, detector-development progresses have been made. Typically, the preferred detectors are those exploiting a maximum of the scattered radiation. Although fast acquisition times can be realized by 1D (Rouquette *et al.*, 2012 ▸) and area detectors (Daniels *et al.*, 2014 ▸) with stroboscopic techniques, they often suffer from limited angular resolution and sensitivity to unwanted stray radiation or background contributions. To achieve angular resolutions at the physical limit, detector concepts using analyser crystals are required. With this concept a decoupling between illuminated sample volume and angular resolution can be achieved. This brings significant drawbacks in measuring only a single point in space at a time, which increases the total measuring time by orders of magnitude. This can be partly compensated by using multi-analyser detectors (MADs) with several channels, each with a single-crystal analyser, and recording diffraction patterns simultaneously with a constant 2θ offset with respect to each other (Hodeau *et al.*, 1998 ▸; Gozzo *et al.*, 2004 ▸; Toraya, 1996 ▸, 2009 ▸; Peral *et al.*, 2011 ▸; Lee *et al.*, 2008 ▸). First proof-of-concept studies using analyser crystals for synchrotron X-ray diffraction experiments were carried out, for example by Buras and Christensen at the DORIS synchrotron in Hamburg (Germany) in 1981 (Buras & Christensen, 1981 ▸). Later implementations by Cox *et al.* at the Cornell High Energy Synchrotron Source (CHESS) and the Brookhaven National Light Source used the same setup (triple-axis diffractometers equipped with an analyser crystal) to record high-resolution diffractograms (Cox *et al.*, 1983 ▸, 1986 ▸; Hastings *et al.*, 1984 ▸). Similarly, Parrish *et al.* used an analyser crystal during experiments at the Stanford Synchrotron Radiation Laboratory to improve angular resolution (Parrish *et al.*, 1985 ▸, 1986 ▸).

Several MAD concepts have been developed in recent years at the Photon Factory (PF) (Toraya *et al.*, 1996 ▸), the European Synchrotron Radiation Facility (ESRF) (Hodeau *et al.*, 1998 ▸; Dejoie *et al.*, 2018 ▸), the Swiss Light Source (SLS) (Gozzo *et al.*, 2004 ▸), the ALBA synchrotron (Peral *et al.*, 2011 ▸) and the Advanced Photon Source (APS) (Lee *et al.*, 2008 ▸). The common design of a typical MAD covers photon energies between 8 and 40 keV. In order to adjust the analyser crystals for the different energies and maximize the transmitted intensities, different levels of complexity have been developed. The simplest design allows a degree of freedom of the secondary collimator and the scintillator detectors with respect to the analyser crystals and the crystals itself (Gozzo *et al.*, 2004 ▸; Hodeau *et al.*, 1998 ▸). More sophisticated solutions were developed (Peral *et al.*, 2011 ▸) or include even individually adjustable analyser crystals (Lee *et al.*, 2008 ▸). These additional degrees of freedom are implemented in order to optimize signal-to-noise ratio and diffracted intensity, since the beam paths on the off-centre crystals and through the channels follow non-linear pathways. A clever approach to optimize these dependencies was proposed by Peral *et al.* (2011 ▸) with a Rowland circle construction. With this setup the disadvantages of beam walk across the components are minimized. A detailed treatment of the instrumental resolution function (IRF) in the presence of mirrors and analysers can be found in the work of Gozzo *et al.* (2006 ▸). However, MAD designs for a broad energy range are limited to maximum photon energies of ∼40 keV. For higher energies, the diffraction angles become smaller and the separation of channels is more challenging and cannot be combined with the adjustment for low photon energies. However, sophisticated *in situ* or *in operando* experiments in materials science or for fundamental research, together with complex sample environments or transmission geometry setups, are frequently limited by absorption. Since the ideal ratio between absorption and sample thickness is reached at μ*R* = 1, with μ being the absorption coefficient and *R* being the sample radius, complex sample environments or geometries can only be realized with increasing photon energy and thus decreasing μ (Cullity & Stock, 2001 ▸; Ehrenberg *et al.*, 2013 ▸, 2019 ▸). These experiments demand higher photon energies with a higher penetration depth or higher transmission capability (Schmitt *et al.*, 2013 ▸; Ehrenberg *et al.*, 2019 ▸).

The challenge with a MAD setup for high photon energies is the small Bragg angles owing to the need to separate the beam paths inside the detector for the direct and the diffracted beams. This channel crosstalk becomes critical for photon energies above a certain limit. As an example, the Si 111 reflection which is used for analyser crystals exhibits a diffraction angle of θ = 1.888° at 60 keV. A typical diffraction pattern usually recorded up to 2θ = 90° using Cu *K*α_1_ radiation shrinks to a 2θ range of only 10.5° for this photon energy of 60 keV. This requires sophisticated shielding, fine adjustment of the analyser crystals and a sufficiently small angular step width of data points. The required step width is determined by the available angular resolution that comes along with the Darwin width of a Si 111 reflection of 2.4 × 10^−4^ degrees. Furthermore, the high penetration capability of the 60 keV primary beam energy requires a proper shielding of scintillator detectors and the collimator path in order to maximize the signal-to-noise ratio.

In order to combine experiments with high penetration capability for highly absorbing materials or *in situ* experiments with high angular resolution and to overcome the limitations of ordinary powder-diffraction experiments, specialized approaches are necessary. One way is to select a photon energy which is high enough to not be limited by absorption edges. In this case, no fluorescence effects limit the diffraction experiments. Such energies typically lie in the range of ∼60 keV and above. On the other hand, the energies should be low enough to be able to be handled with reasonable effort in terms of shielding with tungsten- or tantalum-based materials. A photon energy of 60 keV constitutes a reasonable compromise between the cases described and is generally suited for the majority of specifications.

In this article, we present a ten-channel MAD for the powder-diffraction side station P02.1 (Dippel *et al.*, 2015 ▸; Herklotz *et al.*, 2013 ▸) at the PETRA III storage ring of the Deutsches Elektronen-Synchrotron (DESY) in Hamburg, Germany. This side station operates with an undulator as a radiation source at a fixed energy of 60 keV and is dedicated to high-resolution powder diffraction for material science applications (Liu *et al.*, 2017 ▸; Liu, Knapp, Ehrenberg *et al.*, 2016 ▸; Liu, Knapp, Schmitt *et al.*, 2016 ▸; Hinterstein *et al.*, 2018 ▸), *in situ* and *in operando* studies (Schader *et al.*, 2016 ▸; Geiger *et al.*, 2017 ▸, 2018 ▸; Mgbemere *et al.*, 2017 ▸; Hinterstein *et al.*, 2019 ▸; Riess *et al.*, 2019 ▸; Lee, Shi, Kumar, Hoffman, Etter, Checchia, Winter *et al.*, 2020 ▸; Lee, Shi, Kumar, Hoffman, Etter, Winter *et al.*, 2020 ▸; Choe *et al.*, 2015 ▸), and total-scattering experiments (Yavuz *et al.*, 2015 ▸). Together with proper synchronization with a periodic excitation, this detector can also be used for high-resolution *in situ* and *in operando* stroboscopic measurements on materials under the influence of external stimuli (Liu *et al.*, 2020 ▸; Lee, Shi, Kumar, Hoffman, Etter, Winter *et al.*, 2020 ▸; Choe *et al.*, 2015 ▸). The capability of this detector at beamline P02.1 is shown with measurements on different reference materials [*e.g.* NIST 660a/b (the National Institute for Standards and Technology), NIST 640d, NIST 674b]. The *in situ* behaviour of a highly absorbing piezoceramic and the elucidation of the complex structure of strontium niobium titanate were also determined.

## 2. Technical realization   

The standard detector setup for the beamline is a Perkin Elmer area detector with an active area of 409.6 mm × 409.6 mm and a pixel size of 200 µm^2^. For accurate profile-shape measurements or powder-diffraction measurements at the resolution limit, the ten-channel MAD can alternatively be moved in and rotated around the sample, which itself is mounted in the diffractometer centre. The technical layout of the detector is mainly based on tungsten alloy (Densimet) collimators. Since the beamline is operated at a fixed photon energy, no complex adjustment mechanism is necessary for the analyser crystals. Owing to the high photon energy, the necessary 2θ scanning range is rather small. Therefore, the channels were designed with a separation of just 1° in 2θ from each other. This requires accurate shielding and channel separation for the direct and the diffracted beam in the collimators. To maximize the diffracted intensity and the signal-to-noise ratio, the analyser crystals have to be adjusted accurately. Even miscuts of the crystals and small misalignments can already lead to a significant decrease in diffracted intensity. The alignment of the analyser-crystals’ angle was originally performed via stepper-motor-driven spindles sitting on a slide and acting on a lever arm (Horst *et al.*, 2013 ▸). However, owing to stability reasons and higher repeatability and accuracy, linear piezo actuators with magnetic encoder and reference markers were used to act via lever arms on the crystals. The intensity diffracted by the analyser crystals was detected via scintillation counters (see Fig. S1 in the supporting information).

**Figure 1:**
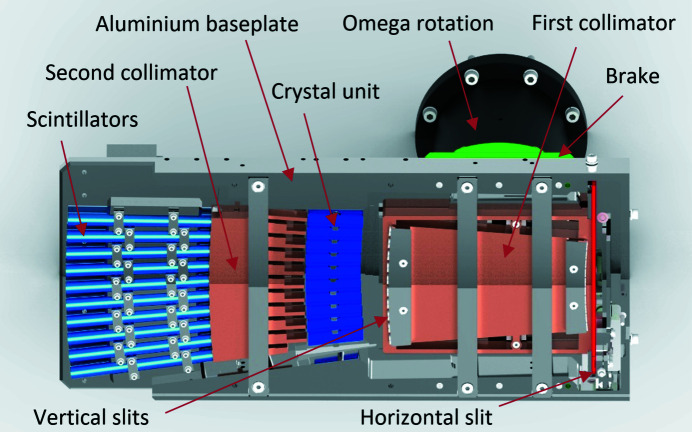
A schematic overview of the complete MAD.

The whole detector sits on a supporting aluminium baseplate where all components can be pre-aligned in machined seats (Fig. 1 ▸). The main components are first collimator unit, crystal unit, second collimator unit and supporting aluminium baseplate with scintillator holders. The first and second collimator units are machined with spark erosion from a monolithic block of tungsten alloy (Densimet) to avoid crosstalk between the individual channels and to block fluorescence that is created along the beam path (Fig. S2). Although the incoming primary beam is highly collimated, it turns out that fluorescence is a serious issue, especially for the channels in the lower part of the detector close to the primary beam path. The individual channels have an acceptance angle of 3.27° that can be further narrowed down with slits on the entrance and exit of the first collimator to 0.73°. These slits can also be replaced by custom-made pin diodes for initial alignment (Fig. S3). In front of the detector entrance, two vertical blades act as a horizontal slit to limit the accepted beam in the plane perpendicular to the diffraction plane. The whole setup can be rotated by a stepper motor (omega rotation) for initial angular alignment and the post with the detector attached is finally being blocked by a brake.

**Figure 2:**
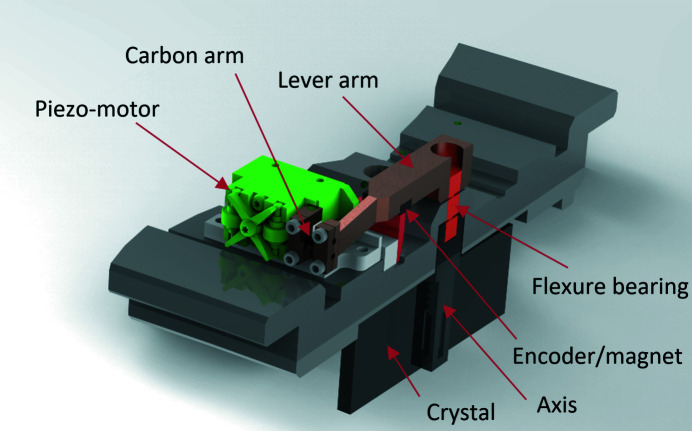
A detailed view of a single piezo-motor-based crystal-alignment module.

**Figure 3:**
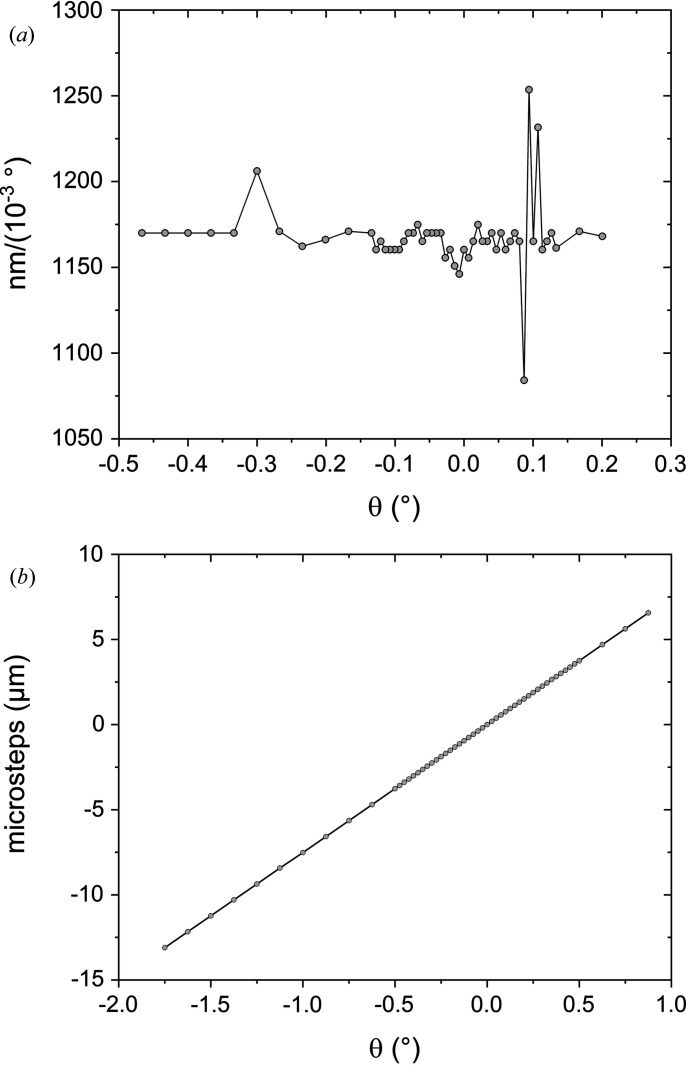
Plots of (*a*) piezo-motor reproducibility versus analyser-crystal axis position and (*b*) piezo-motor microsteps versus analyser-crystal deflection, measured with an autocollimator.

The analyser crystals are 55 mm-long Si(111) crystals and all axes of rotation sit in a common Densimet block exactly around the circumference of the detector circle. To ensure high accuracy and reproducibility of the rotation, and since the required angular range is only about ±0.5°, a flexure hinge bearing was used for the axes (Fig. 2 ▸). One half axis is glued to the front side of the crystal with an opening large enough to let the beam pass through. The second half axis has a lever arm mechanism that is elastically fixed to a linear piezo-motor drive. Both half axes are fitted into the flexure hinge. The elastic connection between the lever arm and the linear piezo drive is made of carbon-fibre-enforced polymer. The linear piezo motor has a magnetic encoder system with reference position. The linear resolution per microstep was chosen to be 7.8 nm which transfers into 2.5 × 10^−5^ degrees. Fig. 3 ▸(*a*) shows the reproducibility of the crystal angle measured with an interferometer versus piezo-motor microsteps. These results over a tilting range of 2.5° show an average value of ∼1175 nm (10^−3^ degrees)^−1^, which results in ∼150 steps (10^−3^ degrees)^−1^. The maximum deviation is ∼100 nm (10^−3^ degrees)^−1^. For the linear plot of microsteps versus analyser-crystal deflection, this deviation is negligible and the setup shows a smooth straight line [Fig. 3 ▸(*b*)].

**Figure 4:**
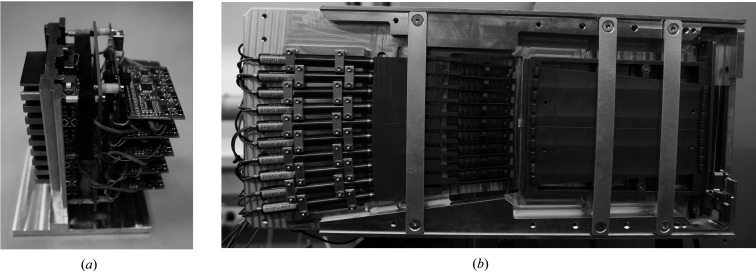
(*a*) An analyser block with electronics and (*b*) the MAD mounted on a diffractometer with the protective cover removed.

The positioning of each analyser crystal is controlled by custom-built electronic boards [Fig. 4 ▸(*a*)] which allow a closed-loop regulation for the piezo motor in a range down to the resolution limit, which corresponds to a resolution of 1.6 × 10^−5^ degrees for the crystal. A ten-channel multiplexer circuit connects each piezo-motor channel via USB or serial port to the remote computer. Comprehensive tests with the direct beam at beamline P02.1 with 60 keV showed that even small corrections in the closed-loop mode can be seen in the diffracted-beam profile. To avoid this, we used the closed-loop positioning system only for the primary adjustment procedure of the crystal. Once the maximum count rate is reached, the proportional–integral–derivative (PID) controller can be switched off and the piezo motor is forced via a command to settle down into a ‘parking position’. The position of the motor is held by mechanical clamping and the electronic voltage supply can be switched off.

## 3. Performance   

The performance of the MAD was determined by measurements of the attenuated primary beam and by measurements of commercially available powder X-ray diffraction standards obtained by NIST. The long-term stability of each channel regarding zero shift and integrated intensities was determined by primary beam measurements and can be found in the supporting information.

**Figure 5:**
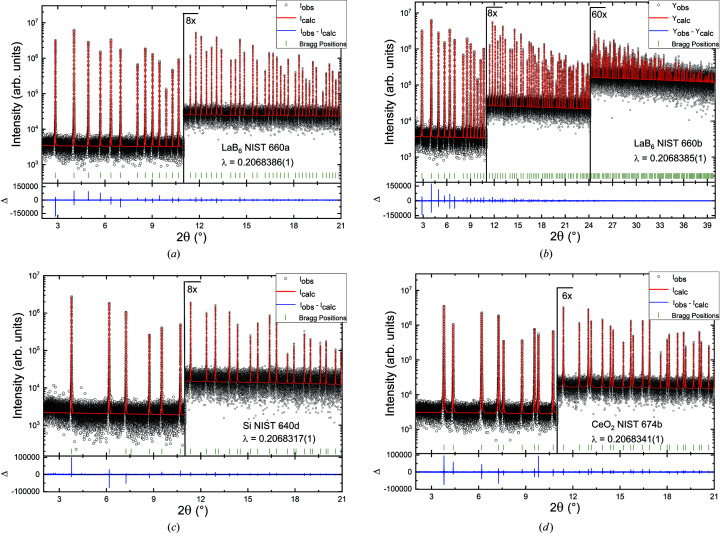
Powder X-ray diffraction measurements with the MAD (circles) and corresponding Pawley refinements (red line) of NIST standards: (*a*) LaB_6_ NIST 660a, (*b*) LaB_6_ NIST 660b, (*c*) silicon NIST 640d and (*d*) CeO_2_ NIST 674b. Difference curves below each diffraction pattern are enhanced.

For the determination of the IRF, several NIST powder X-ray diffraction reference standards were measured: LaB_6_ (NIST 660a, NIST 660b), silicon (NIST 640d) and CeO_2_ (NIST 674b). The measurements were taken on different days. All measurements were performed in a continuous scan mode (sweep mode) for the 2θ circle (see the supporting information) with a step width after re-binning of 2.4 × 10^−4^degrees. Measurements of the individual channels during one run were subsequently merged into a single diffraction pattern. Pawley refinements (Pawley, 1981 ▸) of these measurements made with the program *TOPAS* (Coelho, 2018 ▸) can be found in Figs. 5 ▸(*a*)–5 ▸(*d*).

**Figure 6:**
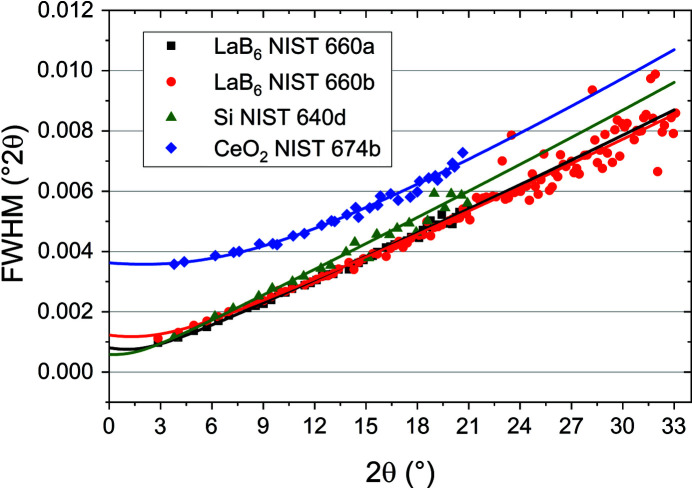
FWHM determined for individual reflections of LaB_6_ NIST 660a (squares), LaB_6_ NIST 660b (circles), silicon NIST 640d (triangles) and CeO_2_ NIST 674b (diamonds). The corresponding lines were calculated using the Thompson–Cox–Hasting parameters, which were determined from Pawley refinements.

Refined parameters from all Pawley fits can be found in Table 1 ▸. For all refinements the Thompson–Cox–Hastings pseudo-Voigt approach (Thompson *et al.*, 1987 ▸) for modelling the 2θ-dependent reflection profiles was used, plus the model of Finger *et al.* (1994 ▸) in order to account for the asymmetric axial divergence effect (the algorithm models the shifts and low-angle tails for peaks below ∼2θ = 30°). During the process of refinement, different axial divergence models were initially tested and it turned out that the ‘simple axial model’ implemented in the *TOPAS* software could be used in order to obtain an adequate modelling of the asymmetric reflection profiles. However, by changing to the model of Finger *et al.* (1994 ▸) with parameters fixed to their physical values (*L* = 551.33 mm, *S* = 0.8 mm, *H* = 2.65 mm) a slightly improved fit could be obtained in terms of a flatter difference curve and a reduced weighted profile *R* factor [residual factors and the goodness of fit (GoF) are used as defined in the *TOPAS* program (Coelho, 2018 ▸)]. For each Pawley refinement, the wavelength, two background coefficients and five Thompson–Cox–Hasting parameters were refined. The quality of the obtained IRF was verified by plotting these functions together with the full width at half-maxima (FWHM) determined by pseudo-Voigt fits of individual reflections of all reference materials (Fig. 6 ▸). All the determined IRFs are in good agreement with the individually fitted reflection widths and behave as described by Masson *et al.* (2001 ▸).

Although the obtained parameters and fits are almost in perfect accordance with expected values, we note that the values for the GoF as well as the (weighted) profile *R* values (even the background-corrected ones) are much higher than one would expect from the appearance of the refinements. These high *R* values can be explained by taking the low background into account, which is in fact flat over the entire measurement range, but showing a rather high scattering, the reason of which could not be identified. When normalizing the highest reflection in the diffraction pattern of Fig. 5 ▸(*a*) to 100, the highest average background value is ∼0.07 ± 0.04 for low angles and decreases linearly to 0.02 ± 0.02. Considering the high photon energy and hence the greater difficulties in controlling stray radiation, the signal-to-background ratio is excellent. With very sharp reflections, even for conservative calculations, this results in just 1% of the data points containing information about reflections. Therefore, the relative scattering of the background data points contributes significantly to the seemingly inferior refinement parameters.

## 4. Materials science case studies   

In the following, two scientific examples are shown that demonstrate the capabilities of the MAD at the P02.1 beamline. Firstly, we provide a case study using strontium niobate titanate (Sr_3_TiNb_4_O_15_, STN) and strontium niobate zirconate (Sr_3_ZrNb_4_O_15_, SZN). We demonstrate that former unresolved structural features can be investigated in complex crystal structures. The combination with the 2D detector illustrates the capabilities for the identification of secondary phases in functional materials. We further provide a second case study using 0.6 BaZr_0.2_Ti_0.8_O_3_–0.4 Ba_0.7_Ca_0.3_TiO_3_ (BCZT) and an *in situ* stroboscopic investigation of the field-induced processes. This case study demonstrates the need for a combination of high photon energies for *in situ* or *in operando* experiments in transmission geometry with high angular resolution for functional materials with phase coexistences. The weak unit-cell distortions in BCZT can only be resolved with the MAD. At the same time, the combination with the stroboscopic data-acquisition setup allows time resolutions in the range of microseconds (Choe *et al.*, 2015 ▸).

## 5. Structural characterization of STN and SZN   

### 5.1. Experimental   

Data were collected at a wavelength of λ = 0.2074426 (4) Å. Two-dimensional data were collected with a 16-inch (∼409.6 mm) 2D flat panel detector of the XRD 1621N ES Series (PerkinElmer) with 2048 × 2048 pixels and a pixel size of 200 µm^2^. The sample distance was 2513 mm in order to achieve high resolution. To meet a high signal-to-noise ratio, the exposure time was 60 s. Details about the setup can be found elsewhere (Herklotz *et al.*, 2013 ▸).

High-resolution data were collected with the MAD in the range 0.5° ≤ 2θ ≤ 12.5°. In order to have high statistics and an ideal signal-to-noise ratio even for low-intensity reflections, the full pattern was merged from 3° stretches of every channel (see the supporting information; Lee, Shi, Kumar, Hoffman, Etter, Checchia, Lemos da Silva *et al.*, 2020 ▸). This results in high counting statistics in the angular range 2.5° ≤ 2θ ≤ 10.5°. In the low and high angular ranges and depending on the 2θ range, only one single pattern or two patterns were measured. Data were collected with an angular step width of Δ2θ = 0.0005° and an exposure time of 7.5 s per point.

Rietveld refinement was performed with the program package *FullProf* (Rodríguez-Carvajal, 1993 ▸). The structure models consisted of three phases of STN. The main phase, Sr_3_Nb_4_TiO_15_, crystallizes with space group *Pna*2_1_ (Whittle & Schmid, 2014 ▸; Whittle *et al.*, 2017 ▸). Additionally, two impurity phases were identified. A cubic perovskite phase of SrNb_0.8_Ti_0.2_O_3_ with space group *Pm* 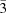 *m* and a complex phase Sr_5_Nb_4_TiO_17_ with space group *Pnnm* (Drews *et al.*, 1996 ▸). The instrumental broadening was determined by a Rietveld fit of a high-resolution measurement recorded at ambient temperature of the standard reference material LaB_6_ (SRM 660a, NIST) for X-ray measurements. The profile function was described using the Thompson–Cox–Hastings pseudo-Voigt model (Thompson *et al.*, 1987 ▸). Lattice parameters, background, scale factors, zero shift for the MAD data and the overall Debye–Waller factor were refined. All other structural information such as atomic positions or individual Debye–Waller factors were kept from literature values. The refinement with the MAD and 2D data was performed simultaneously with a single structure model of three phases in order to combine the high statistics from 2D and the high angular resolution from the MAD.

### 5.2. Results and discussion   

**Figure 7:**
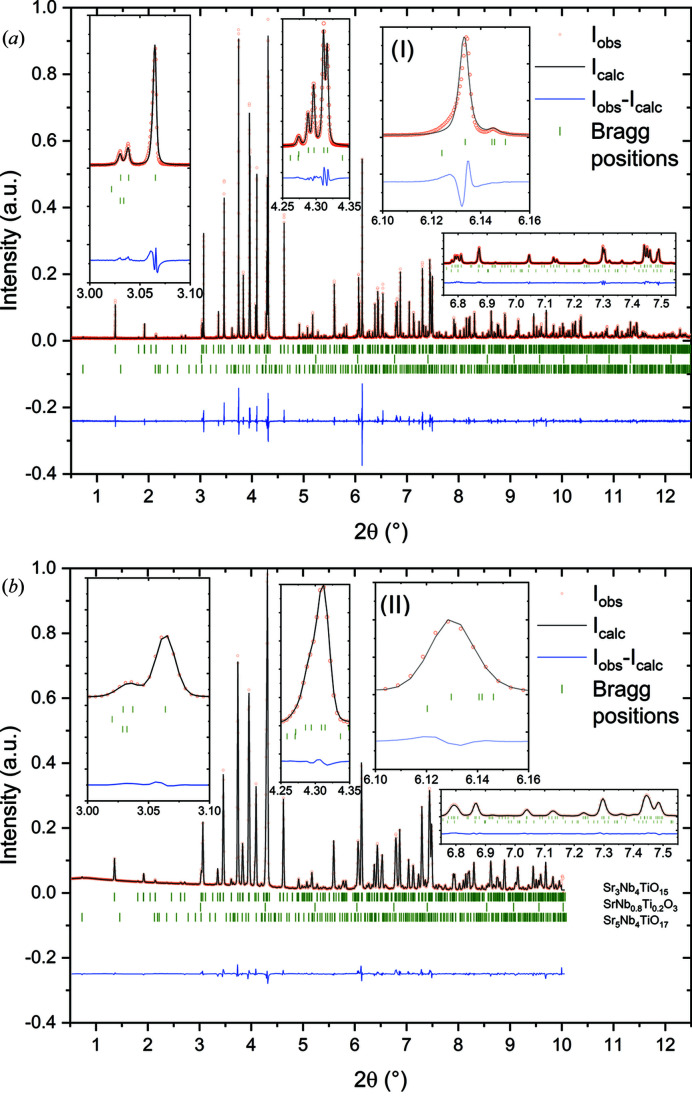
Rietveld refinements with X-ray diffraction patterns of STN, measured with (*a*) the high-resolution MAD and (*b*) the 2D Perkin Elmer detector in high-resolution mode at a sample detector distance of 2513 mm. The diffraction data reveal two impurity phases: SrNb_0.8_Ti_0.2_O_3_ [1.02 (8)%] and Sr_5_Nb_4_TiO_17_ [1.45 (19)%]. The insets show magnifications of a range of individual reflections. The 004 reflection in inset (I) of the high-resolution MAD data reveals an asymmetry, which may result from stacking faults along 00*l*, which can only be detected with the highest angular resolution and not with the 2D detector as shown in inset (II). Red dots indicate measured intensities, black lines indicate the calculated diffraction pattern from the structure model, blue lines indicate the difference between measured and calculated intensities, and green tick marks indicate reflection positions of the respective phases. λ = 0.2074426 (4) Å.

Fig. 7 ▸ shows a comparison of the X-ray diffraction patterns of STN. While Fig. 7 ▸(*a*) shows the high-resolution diffraction pattern collected with the MAD, Fig. 7 ▸(*b*) shows the diffraction pattern of the same sample collected with the 2D Perkin Elmer detector at a high-resolution distance to the sample of 2513 mm. The difference plot in Fig. 7 ▸(*b*) demonstrates that the structure model can explain the observed intensities very well. However, the difference plot in Fig. 7 ▸(*a*) exhibits some strong deviations between the calculated and the observed intensities. A closer look at some characteristic reflections with exceptional deviation, plotted in the insets, shows that the deviations originate from profile mismatches. Most prominent is the profile mismatch for the 004 reflection, shown in inset (I) of Fig. 7 ▸(*a*), which cannot be observed with the 2D detector [Fig. 7 ▸(*a*), inset (II)]. This reflection shows a pronounced asymmetry towards lower angles. This form of asymmetry might originate from stacking faults owing to the plate-like structure of the main phase (Estevez-Rams *et al.*, 2003 ▸). Octahedra in subsequent layers of this structure along the *c* axis are constraint to tilt in the opposite sense reducing the likelihood of stacking faults (Whittle *et al.*, 2015 ▸, 2018 ▸; Campbell *et al.*, 2018 ▸). These might, however, originate at possible cation defect sites. A detailed microstructural analysis of this complex material may be able to shed light on a possible mechanism but is beyond the scope of the work presented here.

**Figure 8:**
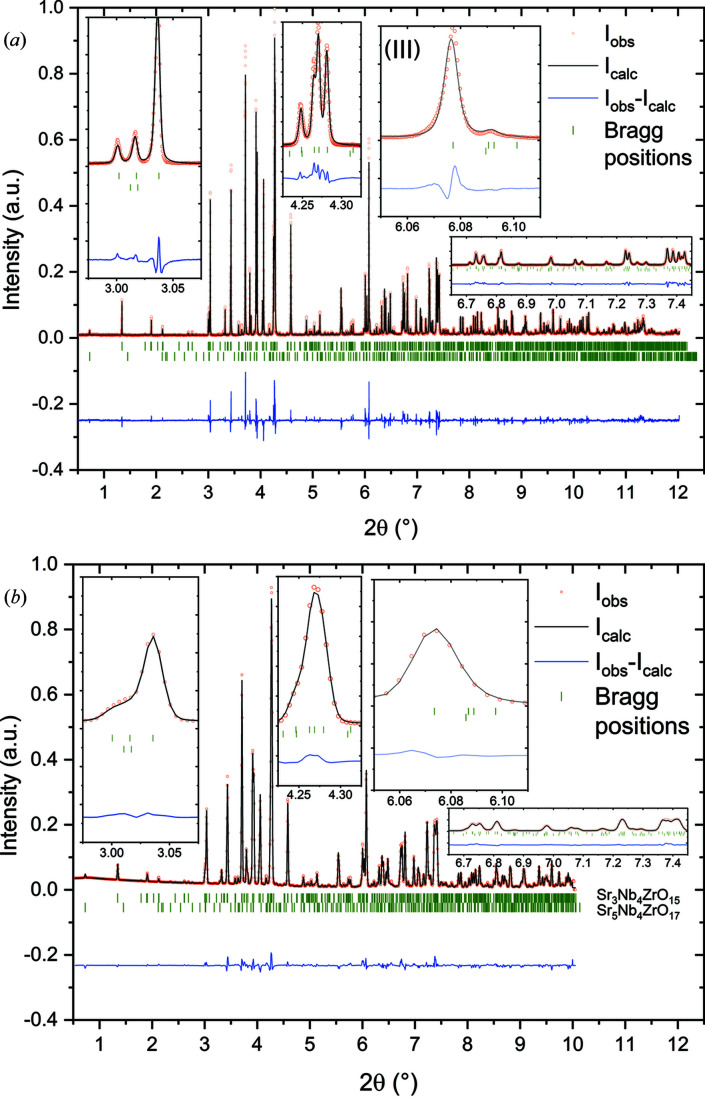
Rietveld refinements with X-ray diffraction patterns of SZN, measured with (*a*) the high-resolution MAD and (*b*) the 2D Perkin Elmer detector in high-resolution mode at a sample detector distance of 2513 mm. The diffraction data reveal one impurity phase: SrNb_0.8_Zr_0.2_O_3_ [4.52 (25)%]. The insets show magnifications of a range of individual reflections. Compared with STN, the asymmetry of the 004 reflection in inset (III) of the high-resolution MAD data is much less pronounced, which indicates only small amounts of stacking faults along 00*l*. Red dots indicate measured intensities, black lines indicate the calculated diffraction pattern from the structure model, blue lines indicate the difference between measured and calculated intensities, and green tick marks indicate reflection positions of the respective phases. λ = 0.2074426 (4) Å.

Fig. 8 ▸ shows a comparison of the X-ray diffraction patterns of SZN, measured with the MAD and the 2D detector. The structure models are similar to STN with slight differences in lattice parameters (Whittle *et al.*, 2020 ▸). The difference curve of the 2D data in Fig. 8 ▸(*b*) shows an agreement similar to that shown in Fig. 7 ▸(*b*), which indicates an equally well fitting structure model. However, a closer look at the MAD data in Fig. 8 ▸(*a*) reveals a significantly worse fit. Although the asymmetry of the 004 reflection cannot be seen very well anymore [Fig. 8 ▸(*a*), inset (III)], the general mismatch of the profile is still the main reason for the difference between observed and calculated intensities. For this sample, the impurity phase content is higher with ∼4.5 wt%. The impurity phase was found to be Sr_5_Nb_4_ZrO_17_ in analogy with STN. In this phase, titanium was substituted by zirconium. However, this phase is not known in the literature nor in any crystal-structure database. The fact that it explains all additional reflections with the modified structure model of Sr_5_Nb_4_TiO_17_ from the literature (Drews *et al.*, 1996 ▸) strongly suggests the existence of this compound. Similar to STN, the proof of existence of this impurity phase was only possible with the combination of MAD and 2D data.

The comparison of the MAD data with the 2D data reveals that, even in the high-resolution position, the 2D detector cannot resolve the fine reflection splitting of STN or SZN. Especially in the range around 2θ = 4.3°, the MAD can resolve five reflections of STN from three phases while the 2D detector only shows a slightly asymmetric single reflection. The combination of MADs and 2D detectors proves to be a strong tool for solving mixed structures from powder-diffraction data.

## 6. *In situ* stroboscopic investigation of the field-induced processes in BCZT   

### 6.1. Experimental   

Data were collected at a wavelength of λ = 0.2072066 (4) Å. The measurements were performed with the custom-built stroboscopic data system MAD-STROBO for time-resolved X-ray diffraction experiments on time scales down to 10 ns (Choe *et al.*, 2015 ▸). Powder X-ray diffraction patterns were taken in transmission geometry with the electric field parallel to the scattering vector. The structural response was measured with the highest possible resolution of the beamline P02.1 with the MAD. The maximum time resolution of the diffraction signal (defined by the width of the time channel) was 10 ns – every 100 adjacent channels were binned to reduce the time resolution to 1 µs.

The investigated sample 0.6 BaZr_0.2_Ti_0.8_O_3_–0.4 Ba_0.7_Ca_0.3_TiO_3_ (Acosta *et al.*, 2015 ▸) has dimensions of 1 mm × 1 mm × 5 mm with electrodes on two opposing long sides that are connected to a high-voltage supply. For this sample, μ*R* = 1.80 at 60 keV, which leads to a transmitted intensity of 2.75%. At usual high-resolution beamlines operating at the highest possible energies of 40 keV, μ*R* = 5.35 affords a transmitted intensity of 0.002%. At 30 keV, which marks the highest possible energy for silicon-based-strip and 2D detectors, μ*R* = 8.40 affords a transmitted intensity of <0.0001%. This shows that these *in situ* high-resolution measurements are only feasible with a combination of high photon energy and a MAD.

### 6.2. Results and discussion   

The aim of this experiment was to investigate the structural changes in 0.6 BaZr_0.2_Ti_0.8_O_3_–0.4 Ba_0.7_Ca_0.3_TiO_3_ (BZT–40BCT) ferroelectric ceramics *in situ*, induced by a unipolar alternating electric field. The BZT–*x*BCT system exhibits a complex phase diagram with rhombohedral, orthorhombic and tetragonal structures below the Curie temperature (Keeble *et al.*, 2013 ▸). The Curie temperatures for the compositions around the phase boundaries of these phases are relatively close to room temperature in the range between 60°C and 90°C. Therefore, the BZT–*x*BCT system is important for electrocaloric applications. However, the unit-cell distortions are weak, which demands for high angular resolution to evaluate crystallographic distortions. Recently, a strong electrocaloric effect was reported for BZT–35BCT (Sanlialp *et al.*, 2015 ▸). This effect peaks around the Curie temperature, which is ∼70°C for the composition BZT–40BCT. In addition, complex polarization dynamics were demonstrated in these materials as a function of temperature, which largely determine the piezoelectric activity (Zhukov *et al.*, 2015 ▸). In order to understand these functional properties and correlate them with the structure of the materials, we performed detailed structural investigations above the Curie temperature.

While ferroelectrics are expected to exhibit cubic structure above the Curie temperature, we recently showed in the system BNT–*x*BT that an applied electric field can induce a paraelectric ferroelectric phase transformation, even several degrees above the Curie temperature (Wang *et al.*, 2014 ▸). In order to investigate this behaviour in BZT–*x*BCT and correlate structural distortions with functional properties, we performed stroboscopic high-resolution powder diffraction above the Curie temperature. The angular resolution at the physical limit allows resolving of even the slightest lattice distortions. Together with the stroboscopic technique, we can thus access fine responses at timescales in the range of microseconds in order to elucidate the electrocaloric phenomenon.

**Figure 9:**
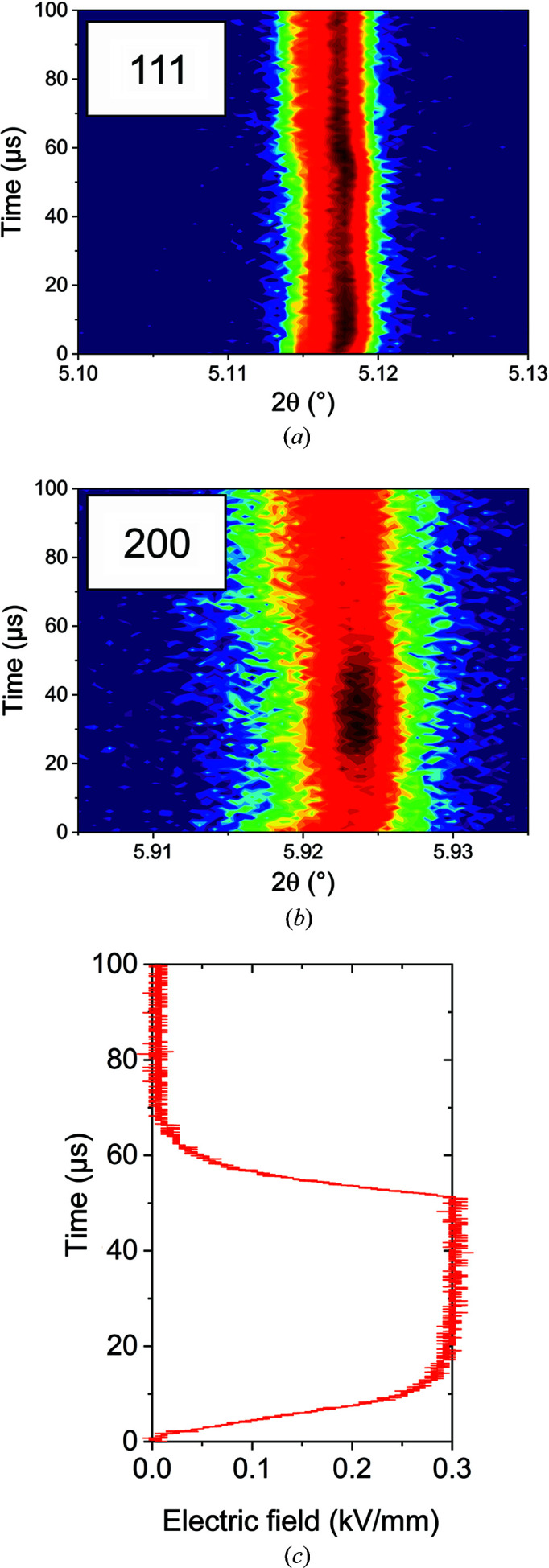
Stroboscopic high-resolution powder diffraction with 10 kHz and 0.3 kV mm^−1^ with a time resolution of 1 µs of BCT–40BZT. Contour plots of the (*a*) 111 and (*b*) 200 reflections and (*c*) the corresponding electric field profile.

Figs. 9 ▸(*a*) and 9 ▸(*b*) show the time-resolved powder-diffraction profiles of the 111 and 200 reflections under a 10 kHz electric field of 0.3 kV mm^−1^. The contour plots directly show pronounced changes to the applied electric field. While the strongest changes of the 111 reflection occur directly after switching on and off the field, the response of the 200 reflection is significantly delayed. The increasing reflection intensities indicate a change of crystal structure or an increase of order [*i.e.* a decrease of distributions of lattice distortions as found in domain structures (Boysen, 2007 ▸)] as a function of the applied electric field. While the 111 reflection can display a splitting owing to orthorhombic (*Amm*2) or rhombohedral (*R*3*m*) distortion, the 200 reflection indicates orthorhombic (*Amm*2) or tetragonal (*P*4*mm*) distortion. In this experiment, the electric field is perpendicular to the incident beam. Therefore, we measure lattice planes that are perpendicular to the electric field and can monitor the piezoelectric effect along the electric field.

Since the applied electric field is a unidirectional force, it induces a preferred orientation and growth of domains with polar directions close to the field direction (Acosta *et al.*, 2016 ▸). The growth of domains with a specific orientation decreases the amount of domain walls and thus the distribution of lattice distortions (Boysen, 2007 ▸). As reported by Jin *et al.* (2003 ▸), fine domain structures in the range of nanometres near phase boundaries can result from conformal miniaturization. With applied field, the authors could show in a relaxor ferroelectric system that the application in such a system leads to discontinuous changes in lattice parameters. This ordering induced by the anisotropy of the electric field together with the very low tolerance level concerning Bragg’s law can result in a change in integrated intensity. This is because it involves an increase in scattering volume that fulfils the diffraction condition. Therefore, the observations in Fig. 9 ▸ can be explained by phase transformations or domain ordering.

Upon field application (*t* = 0 µs), the integrated intensity of the reflections increases with the same slope as the electric field. Therefore, the kinetics of this response is at least faster than 10 µs. In this time range, the electric field may induce a phase transformation or the precipitation of domains. At *t* = 15 µs when the maximum field is reached, the intensity of the 111 reflection decreases again, while the intensity of the 200 reflection increases. Since both intensities are inversely correlated, this indicates a complex structural response of the material.

At *t* = 40 µs, the 200 intensity decreases again even though the maximum electric field is still applied. At the same time, the 111 intensity increases back to the maximum reached at the beginning of the field application. This time range might indicate competing strain mechanisms with resonant elastic responses, especially because the 111 response exhibits an exact symmetrical shape beyond the moment of switching off the field. In contrast to the instant response at the rising edge, the response at the falling edge is significantly delayed. This might indicate resonant vibrations owing to the competing structural strain mechanisms.

**Figure 10:**
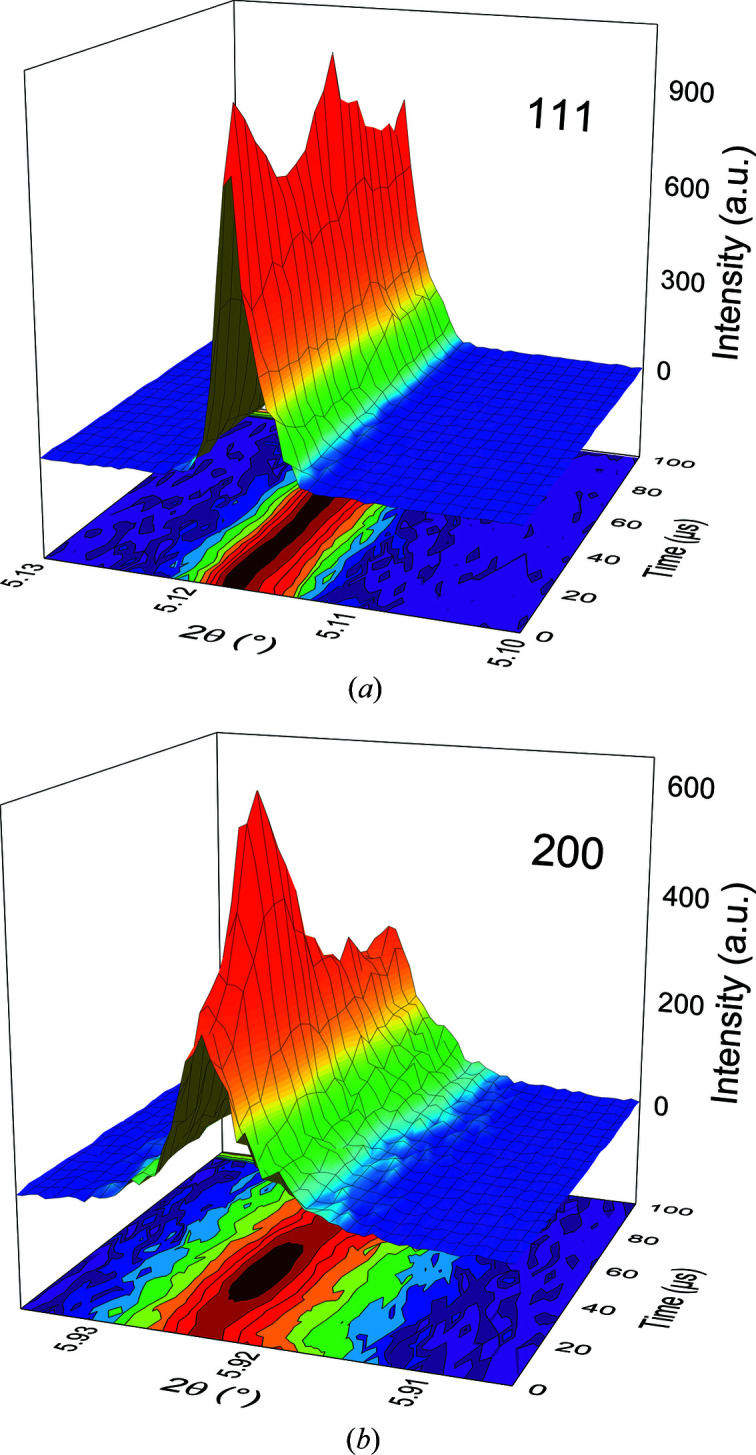
3D contour plots of (*a*) the 111 and (*b*) 200 reflections.

Fig. 10 ▸ depicts the same reflections on 3D contour plots. This representation illustrates the complex and contrarious structural response of the material as a function of time and thus applied electric field. The intensity of the 200 reflection increases dramatically after applying the field and shows pronounced kinetics. At the same time the intensity of the 111 reflection decreases and both intensities follow an opposite behaviour. This again shows the structural connection and points towards a field-induced phase transformation. Since the 200 reflection is significantly broader, it indicates a tetragonal distortion.

## 7. Conclusions   

The structural investigation of STN and SZN reveals that, even in the high-resolution position, the 2D detector cannot resolve the fine distortion of the structure. Some reflections exhibit a fivefold splitting, while the 2D detector only shows a slightly asymmetric single reflection. We demonstrate that it is possible to attain a significant reduction in measuring time with a remarkable resolution using a high-resolution MAD. Stitching together 3° slices of the ten individual channels of the MAD allowed a significant reduction in measuring time for this high-resolution detector and resulted in a good fit, apart from the profile mismatches owing to the real structure of the sample. The fit shows that small quantities of ∼1–2 wt% can still be detected. This also demonstrates the high signal-to-noise ratio and high accuracy in detected intensities. Without the MAD data, it would not have been possible to identify the impurity phases, owing to the strong overlap within the 2D dataset. For the case of SZN, even a formerly unknown compound could be identified. The combination of MADs and 2D detectors proves to be a strong tool for solving mixed structures from powder-diffraction data.

The stroboscopic experiment with applied electric field shows a complex range of responses above the Curie temperature. Only the combination of high angular resolution at the physical limit with the highest brilliance of the synchrotron radiation and a time resolution in the range of microseconds is able to reveal these field-induced processes. This sophisticated experiment demonstrates the possibilities for next-generation materials characterization. The elucidation of competing structural strain mechanisms above the Curie temperature with significantly different time scales are of great importance for the understanding of functional ceramics. These experiments and the MAD become particularly relevant owing to the large absorption of these materials. A detailed characterization of the kinetics of the individual strain mechanisms allow optimizing the efficiency of actuators and electrocaloric applications. Since these complex responses on a microsecond time scale were formerly unknown, this characterization technique has an extraordinary impact on research and development of functional piezoceramics.

## Supplementary Material

Supporting information. DOI: 10.1107/S1600577520013223/co5144sup1.pdf

## Figures and Tables

**Table 1:** An overview of the determined parameters from Pawley refinements Residual factors and the GoF were obtained as defined in the *TOPAS* program (Coelho, 2018 ▸). Lattice parameters are fixed to the NIST certificate values (certified at 22.5°C). The measurements were performed at 23.0°C.

Material	LaB_6_	LaB_6_	Si	CeO_2_
Standard	NIST 660a	NIST 660b	NIST 640d	NIST 674b
Capillary	Kapton tube, 0.8 mm diameter			
Wavelength (Å)	0.2068386 (1)	0.2068385 (1)	0.2068317 (1)	0.2068341 (1)
Lattice parameter (Å)	4.1569162	4.15689	5.43123	5.411526
*U*	8.04 (10) × 10^−4^	7.72 (8) × 10^−4^	4.90 (31) × 10^−4^	8.05 (34) × 10^−4^
*V*	−1.66 (12) × 10^−5^	−2.08 (11) × 10^−6^	−1.20 (38) × 10^−5^	−6.96 (56) × 10^−5^
*W*	3.92 (34) × 10^−7^	9.72 (4) × 10^−7^	1.61 (110) × 10^−7^	6.18 (22) × 10^−6^
*Z*	0	0	0	0
*X*	2.52 (8) × 10^−3^	2.50 (8) × 10^−3^	17.03 (27) × 10^−3^	11.35 (21) × 10^−3^
*Y*	3.13 (5) × 10^−4^	4.25 (5) × 10^−4^	2.98 (16) × 10^−4^	18.69 (15) × 10^−4^
μ*R* (calculated)†	0.56	0.56	0.01	1.40
*R*_exp_	1.12	1.31	1.67	1.14
*R*′_exp_	1.47	1.89	2.46	1.46
*R*_p_	10.98	13.56	17.49	10.59
*R*′_p_	16.77	23.54	31.09	15.45
*R*_wp_	17.24	20.58	26.39	17.78
*R*′_wp_	22.57	29.59	38.91	22.79
*R*_B_	1.06	1.03	0.72	0.58
GoF (*R*_wp_/*R*_exp_)	15.36	15.67	15.80	15.64
GoF (*R*_B_/*R*_exp_)	0.95	0.79	0.43	0.51

† μ*R* is calculated with the help of https://11bm.xray.aps.anl.gov/absorb/absorb.php assuming a packing fraction of 0.5.
