# Supplementary material for: Multi-analyser detector (MAD) for high-resolution and high-energy powder X-ray diffraction
Source: J Synchrotron Radiat. 2021 Jan 1;28(Pt 1):146–57. doi: 10.1107/S1600577520013223 (PMC7842216; doi:10.1107/S1600577520013223)
Supplement: Supplementary file 1 [file s-28-00146-sup1.pdf]

JOURNAL OF SYNCHROTRON RADIATION

Volume 28 (2021)

Supporting information for article:

Multi-analyser detector (MAD) for high-resolution and high-energy powder X-ray diffraction

Alexander Schökel, Martin Etter, Andreas Berghäuser, Alexander Horst, Dirk Lindackers, Thomas A. Whittle, Siegbert Schmid, Matias Acosta, Michael Knapp, Helmut Ehrenberg and Manuel Hinterstein

S1. Performance analysis of the scintillator/photomultiplier assemblies

The homogeneity of the scintillator/photomultiplier assemblies was confirmed by scanning a 0.1 x 0.1 mm² 60 keV X-ray beam in 0.1 mm steps over the whole front window of the assembly while recording the output of the photomultiplier. All 10 scintillator/photomultiplier assemblies showed good homogeneity and linearity over the full area of the scintillator crystal. As an example, two scans with different incident photon flux are shown in Figure S1.

a) b)

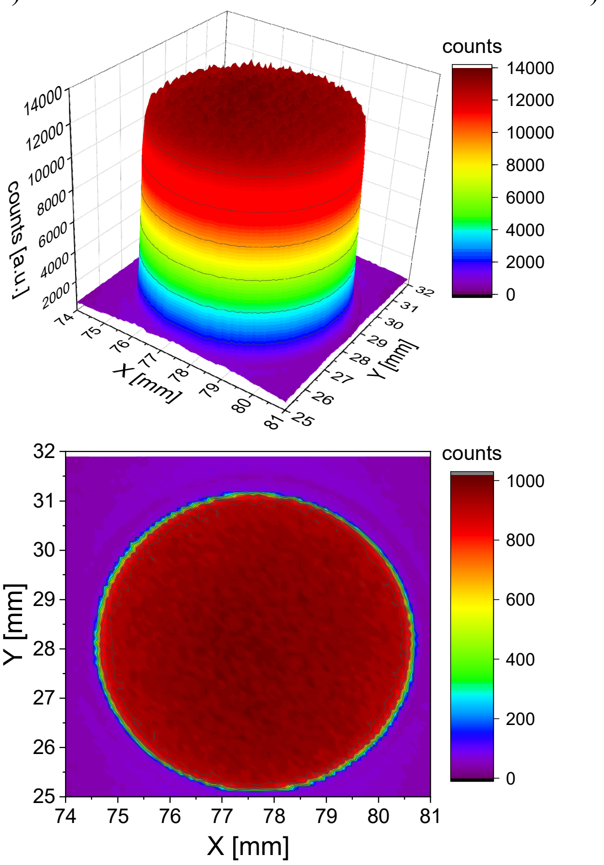

Figure S1 a) 3D plot of the recorded intensity, mapped across a photomultiplier. b) Contour plot of the same photomultiplier at an attenuated incident X-ray photon flux. The experiments were carried out at the BAM beamline at the BESSY synchrotron in Berlin, Germany.

S2. Alignment and calibration of the Multi-Analyser Detector

S2.1. Coarse adjustment via photo diodes

The first collimator block in the MAD assembly is equipped with slots for interchangeable apertures at the entrance and exit of each of the 10 channels (see Figure S2, green and red boxes). The 3 mm thick inserts are machined from Densimet® and define a window of 1 x 5 mm² (Figure S3a). For alignment purposes two additional inserts have been hollowed out and a visible light protected photodiode was installed directly behind the slit window (Figure S3b).

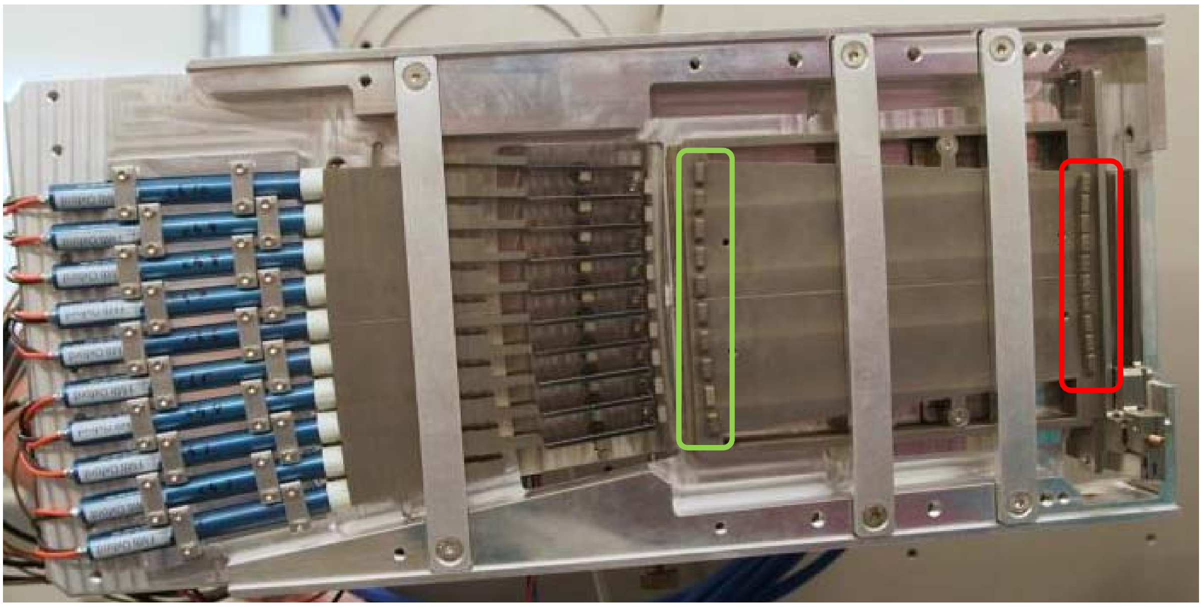

Figure S2 Position of aperture slits at entrance (red) and exit (green) of the first collimator.

a) b)

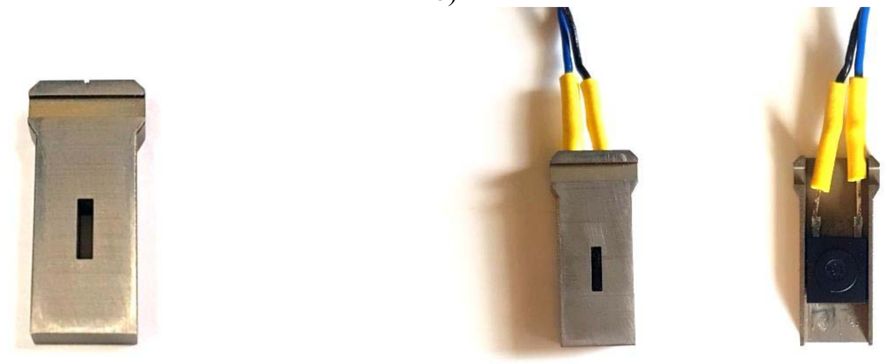

Figure S3 Example of apertures to be inserted into the collimator. a) standard aperture and b) an aperture with integrated photodiode for alignment.

The first step in aligning the MAD is to ensure that all 10 channels are pointing towards the rotational centre of the diffractometer, i.e. the sample position. A diode insert was placed in the front and back of one channel (e.g. channel 1) and the further beampath through the MAD was blocked to prevent oversaturation of the photomultiplier tubes (PMT). While reading the diode currents the diffractometer circle on which the MAD is mounted was scanned. Assuming a "completely misaligned" system only the front diode will record an incoming photon flux when passing through the primary X-ray beam. Since the beam passing through the first diode is blocked by the misaligned collimator, the diode at the end of the channel does not receive any photons. At the diffractometer angle where the maximum current in the front diode is recorded a scan of the pivot angle of the detector is conducted. This is done in order to find a position where also the diode in the back of the channel is hit by X-ray photons. At this new pivot angle of the detector the next scan of the diffractometer circle is conducted. Repeating this procedure one can iteratively find the correct pivot angle of the MAD. To check for consistency and also to account for any manufacturing tolerances of the collimator the two diodes are

then placed in another channel (e.g. channel 10) and the scans repeated. Alternating between channel 1 and 10 the optimal pivot angle can be determined. One last scan of the diodes was performed after the mechanical brake of the pivot rotation was engaged to ensure that the brake did not exert any torsional force onto the detector.

S2.2. Fine adjustment via scan of the attenuated incident beam

After the coarse alignment of the whole detector setup, the analyser crystals need to be adjusted. For this the diode inserts are replaced by proper apertures and the aforementioned protective beamstop is removed. At each of the channel positions, as determined by the diode scans from the coarse alignment (i.e. optimal position of the first collimator), a scan of the corresponding analyser crystal angle is performed using the attenuated primary X-ray beam. These scans are now recorded using the actual scintillator/PMT. After setting the optimal crystal angle the piezo motors where disengaged. Finally a scan of the whole detector is done to test the stability of the positions.

S2.3. Software-based calibration of the rotational center of the diffractometer, determination of incident beam offsets and determination of wavelength

At beamline P02.1, parameters like the exact position of the rotational center of the diffractometer, primary beam offsets in terms of angular and intensity offsets as well as the channel-averaged wavelength are highly dependent on synchrotron and beamline optic parameters. Influencing factors can be the electron beam orbit of the synchrotron or the monochromator angles. These parameters determine the exact position of the beam such as the spatial position as well as the canting of the beam, when it enters the experimental hutch. Therefore, an adjustment and determination of these parameter values has to be done on a weekly or more frequent basis.

For this purpose an automatization routine was written in Python which automatically determines the rotational centre of the diffractometer. This procedure is followed by a measurement and determination of primary beam parameters. A subsequent measurement of a reference material (e.g. LaB_6) is used to determine the instrumental profile and the channel-dependent wavelengths from which an average wavelength then in turn is determined.

The Python program starts with an initial check of possible motor collisions. This means motor positions of motors like the long translation stage of the Perkin Elmer detector are checked and if they are within a “restricted” area, these motors are automatically driven in a predefined save position. This ensures that the MAD cannot collide with any other stages.

In the next step, the script checks if the encoders, e.g. for the diffractometer circle and the monochromator, are active and in closed loop. If a referencing process is required, the program automatically finds the reference position of the diffractometer circles and activates closed loop. At the end of all check-ups, the program creates subfolders in a user directory on the control computer, where the calibration measurements and the obtained parameters will be stored.

The calibration of the rotational center of the diffractometer starts with centering the xyz sample stage sitting on the innermost diffractometer circle. On top of this xyz stage, a capillary spinner with a capillary filled with LaB_6 (reference material NIST 660a or 660b) is mounted. In order to determine the rotational center, absorption measurements of the capillary at four different diffractometer circle positions are made. For this purpose, the program automatically starts the spinning of the capillary, opens the beam shutter and moves a PIPS diode in the incident X-ray beam. Then a xyz sample stage motor is moved vertically/orthogonal to the incident X-ray beam in order to make absorption measurements of the capillary. After this measurement the diffractometer circle is moved clockwise by 90° and the next absorption measurement begins. This is done until all four measurements between 0° and 270° are performed. The program then determines the values of all absorption minima and uses pairs of minima (the one's separated by 180°) in order to calculate and therefore determine the rotational center of the xyz sample stage on the diffractometer.

After centring the capillary with respect to the diffractometer, the diffractometer itself has to be centred with respect to the primary beam. This is again accomplished by a vertical absorption scan with help of the PIPS diode. The horizontal position of the diffractometer is aligned by a respective scan with one of the MAD detector channels and the attenuated primary beam.

After the alignment of the diffractometer, the incident X-ray beam is scanned with each MAD channel in order to obtain the channel-dependent angular and intensity offsets. Since the incident beam has almost a perfect Gaussian profile, the angular offset (= zero shift/error) can be determined by the determination of the profile center and the intensity sensitivity offset (= channel efficiency) is given by the integrated area of the profile. These parameters are automatically evaluated by a fitting routine within the Python program and are then written to a special configuration file, which can be used to correct all subsequent measurements.

As last procedure, the program automatically tries to determine the channel-averaged wavelength of the incident monochromatic X-ray beam. For this purpose, the program moves the LaB_6 capillary and the beam stop back into the beam and removes the absorber plates. Then in each of the ten MAD channels a scan of the LaB_6 322 reflection is performed. The LaB_6 322 reflection is chosen since it is almost free of any axial divergence contribution and has, therefore, a symmetric peak shape. However, before the Python script starts the analysis of the reflections, it normalizes the measurements with the monitored incident flux, fixes the constant channel offset and corrects with the values determined from the incident beam for the channel-dependent zero shift and channel efficiency. Knowing the precise lattice parameters from the LaB_6 reference material and the position of the measured LaB_6 322 reflection, the program calculates the wavelength as measured with each channel. At the end, the program determines the average of all measured wavelength values.

S3. Long-term stability of the Multi-Analyser Detector

The long-term stability of the Multi-Analyser Detector and especially the positional stability of the analyser crystals was examined by performing different scans of the incident X-ray beam in all 10

channels over a period of approximately 3 days. Each channel shows a good stability of the angular offset over time (Figure S4a), which can be determined as the median of the Gaussian incident beam profile as well as a good stability of the intensity efficiency (Figure S4b), which is determined as the integrated area of the Gaussian profile.

a) b)

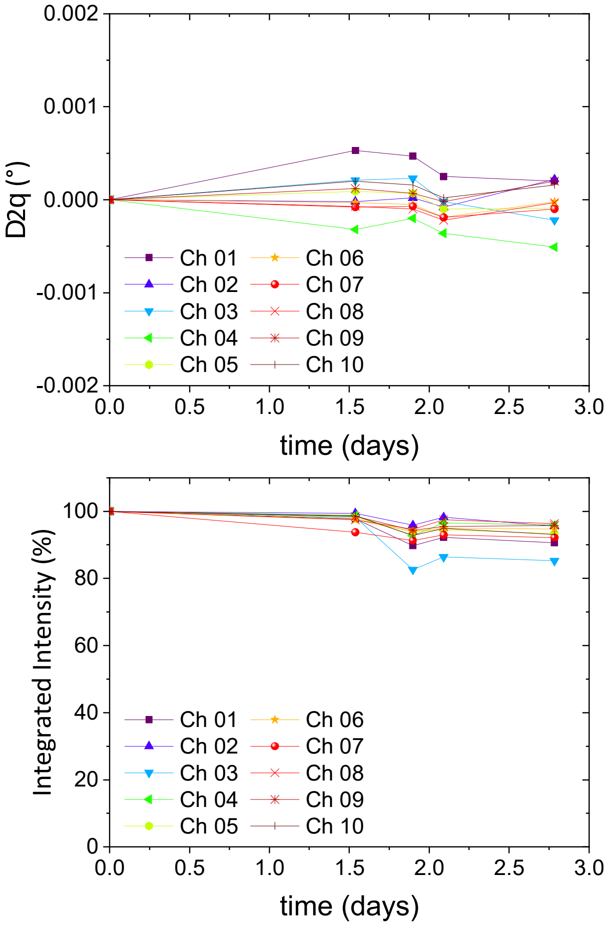

Figure S4 a) Plots of the determined channel-dependent angular offset (= zero shift/error) over time and b) the channel-dependent integrated intensity efficiency over time. Both values are extracted from the scans of the Gaussian primary X-ray beam profile.

S4. Scan modes, data collection and data treatment / software details

S4.1. Scan / Measurement modes

For the measurements with the Multi-Analyser Detector two scan / measurement modes are available: step scans or continuous / sweep scans. In the step scan mode, measurements are performed stepwise, which means that the diffractometer motor drives the Multi-Analyser Detector to a certain 2θ position and holds it there until the data collection at this step is done. Hereafter it drives to the next step position and proceeds with data collection. In the continuous or sweep scan mode the diffractometer drives at constant (slow) velocity over the desired 2θ range and data points are taken after a software triggered encoder readout in certain steps, while the detector moves. For fast measurements the

continuous scan mode is preferred, since no time for repositioning is needed. For long measuring times the step scan mode is necessary when the continuous driving speed is below the lower limit of the goniometer.

S4.2. Data collection strategies

In principal, two different data collection strategies exist. In the first strategy all channels collect data in one particular 2θ range. This requires overlapping 2θ ranges. Since the channels are separated by about 1 degree, this means that above and below this range of overlapping data points a clipping of 9 degrees exists, which is directly related to a required longer measurement time. However, this strategy guarantees good statistics, since all ten channels have seen the same data point in the particular 2θ range and the overlapping region will be summed up to a single pattern.

The second collection strategy exhibits lower quality in statistics but shorter measuring time, since a single data point is seen by only one channel. For this strategy a single Multi-Analyser Detector channel collects data points within about a 1° 2θ interval. With all ten channels together this leads to a collected data range of 10 degrees. These ten individual 1° ranges are subsequently stitched together in order to result in a single pattern. Overlapping angular ranges can be used for normalization. See also (Lee, Shi, Kumar, Hoffman, Etter, Checchia, Silva *et al.*, 2020). Although measurement time will be shorter compared to the first strategy, intensity normalization and 2θ corrections of the individual channels is of much more importance. In principle also a range of less than 1 degree is possible. However, this results in a final pattern with gaps. The values for calibration are estimated with standard reference materials like SRM 660b and then used for a later one measurement.

S4.3. Data treatment / software details

At beamline P02.1, measurement data with the Multi-Analyser Detector can be collected either by using the data acquisition and beamline control software ONLINE or by using Python programs which either work in sweep scan mode or in step scan mode. Data files are stored as ASCII files either in fio or in xy format. In both file types the 2θ positions of the diffractometer, obtained from encoder values as well as integrated counts are saved.

For the data collection with overlapping regions, summation of all ten channels can be performed using a Python program, which first normalizes the intensities of the data. To calibrate the intensities, the primary beam is measured with each channel and then the peak intensity value is used as a correction factor for the individual channels. The primary beam position is used to determine the angular spacing between the channels as well as zero offset of the detector. The 2θ scale and the corresponding intensity readings for each channel are linearly interpolated from the encoder readout to equidistant steps and accordingly weighted intensities. The re-binned channels are then summed up to a single merged data file.

In order to judge how much the software-based data treatment and summation process influences the results, the full width half maximum (FWHM) of the NIST 660a LaB_6 322 reflection was determined

by a Pseudo-Voigt function in TOPAS for each channel and is shown together with the finally merged data set in Figure S5.

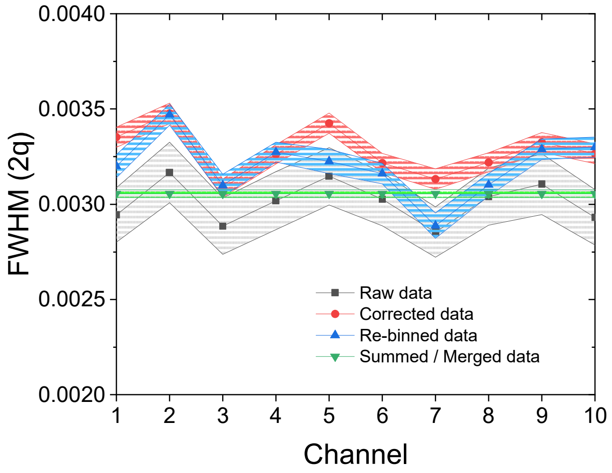

Figure S5 Plots of the full width half maximum (FWHM) of the NIST 660a LaB_6 322 reflection for all ten channels at different data treatment stages determined by a Pseudo-Voigt function in TOPAS. Raw data is the data as collected by the detector (black curve), corrected data means data sets which were corrected for channel-dependent zero shift, channel offset and normalized by the channel-dependent intensity sensitivity (red curve) and re-binned data means data sets where all data sets were re-binned to equidistant x-axis points (blue curve). In addition the FWHM of the summed / merged data set is shown (green curve).

From Figure S5 it is clear that the intensity normalization in the correction process has an influence on the channel-dependent FWHMs, since shifts in 2θ space cannot affect the FWHM. On the other hand the re-binning has only a relatively small effect. The impact of re-binning is negligible due to oversampling. Interestingly, the observable broadening in FWHM is almost corrected when all diffraction patterns are summed up into a single pattern. We note that for a longer integration time per data point, the normalization would not have such a crucial impact on the FWHM, since deviations in intensity are smoothed out over time.

S5. Measurements of reference materials

The measurements of the reference materials LaB_6 (NIST 660a, NIST 660b), silicon (NIST 640d) and CeO_2 (NIST 674b) are attached as supplementary materials to this publication.

S6. Rietveld refinement of reference materials

In addition to the Pawley refinements for the determination of the Instrumental Resolution Function in the main manuscript, Rietveld refinements (Rietveld, 1969) were performed on the same measured data of the reference materials LaB_6 (NIST 660a, NIST 660b), silicon (NIST 640d) and CeO_2 (NIST 674b). Refined parameters determined with the program TOPAS (Coelho, 2018) from all Rietveld fits (not shown here as they look identical to the Pawley fits in Figure 6 in the main manuscript) can be

found in Table S1. Please be aware that two NIST certificates exist for 674b from 2007 and 2017 (the latter was chosen for the present refinements).

Table S1 Overview of determined parameters from Rietveld refinements. Residual factors and the Goodness-of-Fit (GoF) are obtained as defined in the TOPAS program (Coelho, 2018). Measurements were performed at 23.0°C.

Material	**LaB6**	**LaB6**	**Si**	**CeO2**
Standard	**NIST 660a**	**NIST 660b**	**NIST 640d**	**NIST 674b**
Capillary	Kapton tube, 0.8 mm diameter			
Wavelength (Å)	0.2068386	0.2068385	0.2068317	0.2068341
Zero Shift (°2θ)	0	0	0	0
Lattice parameter (Å)	4.1569162	4.15689	5.43123	5.411526
U (10^{-4})	7.94	7.76	4.85	7.99
V (10^{-5})	-1.66	-0.23	-1.16	-6.84
W (10^{-6})	0.41	0.10	0.18	6.13
Z	0	0	0	0
X (10^{-3})	2.83	2.82	17.07	11.32
Y (10^{-4})	3.00	4.11	2.94	18.71
Atomic parameters	La: Biso = 0.366(3) Å² B: z = 0.2005(3) Biso = 0.206(18) Å²	La: Biso = 0.370(1) Å² B: z = 0.2001(3) Biso = 0.241(13) Å²	Si: Biso = 0.399(5) Å²	Ce: Biso = 0.217(3) Å² O: Biso = 0.435(19) Å²
µR (calculated)*	0.56	0.56	0.01	1.40
Rexp	1.12	1.31	1.67	1.14
Rwp	17.29	20.60	26.44	17.80
RB	1.56	1.42	2.45	0.82
GoF (RB / Rexp)	1.39	1.08	1.47	0.72

* µR is calculated with the help of https://11bm.xray.aps.anl.gov/absorb/absorb.php assuming a packing fraction of 0.5.

In the Rietveld refinement the more accurate wavelength from the Pawley fits were used and fixed, since even small drifts in wavelength over time can be seen in the high resolution data. Also, zero-shift

was fixed and the nominal lattice parameters from the certificates were used. The profile parameters show no considerable difference to the Pawley fit.

Similar to the Pawley refinements the Thompson-Cox-Hastings pseudo-Voigt approach (Thompson et al., 1987) for modelling the 2θ-dependent reflection profiles was used, plus the model of Finger et al.(Finger et al., 1994) in order to account for the asymmetric axial divergence effect.

For each Rietveld refinement two background coefficients, five Thompson-Cox-Hasting parameters and a scale factor were refined. For the refinement of the LaB_6 pattern, two additional isotropic displacement parameters and the z-coordinate of the B atom were allowed to vary, which resulted in a total number of 11 refined parameters. For the refinement of the Si pattern only one additional isotropic displacement parameter was allowed to vary, resulting in a total number of 9 parameters. And for the refinement of the CeO_2 pattern, two individual isotropic displacement parameters for each atom were allowed to vary, resulting in a total number of 10 refined parameters.

References

Acosta, M., Khakpash, N., Someya, T., Novak, N., Jo, W., Nagata, H., Rossetti Jr., G. A. & Rödel, J. (2015). *Phys. Rev. B.* **91**, 104108.

Acosta, M., Schmitt, L. A., Cazorla, C., Studer, A. J., Zintler, A., Glaum, J., Kleebe, H.-J., Donner, W., Hoffman, M., Rödel, J. & Hinterstein, M. (2016). *Sci. Rep.* **6**, 28742.

Billinge, S. J. L. (2019). *Philos. Trans. R. Soc. A Math. Phys. Eng. Sci.* **377**, 413.

Boysen, H. (2007). *J. Phys. Condens. Matter*. **19**, 275206.

Buras, B. & Christensen, F. (1981). *Hasylab Annual Reports*, Vol. p. 101.

Campbell, B., Howard, C. J., Averett, T. B., Whittle, T. A., Schmid, S., Machlus, S., Yost, C. & Stokes, H. T. (2018). *Acta Crystallogr. Sect. A Found. Adv.* **74**, 408–424.

Choe, H., Gorfman, S., Hinterstein, M., Ziolkowski, M., Knapp, M., Heidbrink, S., Vogt, M., Bednarcik, J., Berghäuser, A., Ehrenberg, H. & Pietsch, U. (2015). *J. Appl. Crystallogr.* **48**, 970–974.

Coelho, A. A. (2018). *J. Appl. Crystallogr.* **51**, 210–218.

Cox, D. E., Hastings, J. B., Cardoso, L. P. & Finger, L. W. (1986). *Mater. Sci. Forum*. **9**, 1–20.

Cox, D. E., Hastings, J. B., Thomlinson, W. & Prewitt, C. T. (1983). *Nucl. Instruments Methods Phys. Res.* **208**, 573–578.

Cullity, B. D. & Stock, S. R. (2001). Elements of X-ray Diffraction Prentice Hall.

Daniels, J. E., Cozzan, C., Ukritnukun, S., Tutuncu, G., Andrieux, J., Glaum, J., Dosch, C., Jo, W. & Jones, J. L. (2014). *J. Appl. Phys.* **115**, 224104.

Dejoie, C., Coduri, M., Petitdemange, S., Giacobbe, C., Covacci, E., Grimaldi, O., Autran, P.-O., Mogodi, M. W., Šišak Jung, D. & Fitch, A. N. (2018). *J. Appl. Crystallogr.* **51**, 1721–1733.

Dippel, A., Liermann, H., Delitz, J. T., Walter, P., Schulte-Schrepping, H., Seeck, O. H. & Franz, H. (2015). *J. Synchrotron Radiat.* **22**, 675–687.

Drews, A. R., Wong-Ng, W., Roth, R. S. & Vanderah, T. A. (1996). *Mater. Res. Bull.* **31**, 153–162.

Ehrenberg, H., Hinterstein, M., Senyshyn, A. & Fuess, H. (2019). *International Tables for Crystallography, Vol. H*, Vol. pp. 174–188. International Union of Crystallography.

Ehrenberg, H., Knapp, M., Hartmann, T., Fuess, H. & Wroblewski, T. (2000). *J. Appl. Crystallogr.* **33**, 953–957.

Ehrenberg, H., Senyshyn, A., Hinterstein, M. & Fuess, H. (2013). *Modern Diffraction Methods*, Vol. edited by E.J. Mittemeijer & U. Welzel, pp. 491–517. Weinheim, Germany: Wiley-VCH Verlag GmbH & Co. KGaA.

Estevez-Rams, E., Leoni, M., Scardi, P., Aragon-Fernandez, B. & Fuess, H. (2003). *Philos. Mag.* **83**, 4045–4057.

Fauth, F., Peral, I., Popescu, C. & Knapp, M. (2013). *Powder Diffr.* **28**, S360–S370.
Finger, L. W., Cox, D. E. & Jephcoat, A. P. (1994). *J. Appl. Crystallogr.* **27**, 892–900.
Fitch, A. N. (2004). *J. Res. Natl. Inst. Stand. Technol.* **109**, 133.
Geiger, P. T., Clemens, O., Khansur, N. H., Hinterstein, M., Sahini, M. G., Grande, T., Tung, P., Daniels, J. E. & Webber, K. G. (2017). *Solid State Ionics*. **300**, 106–113.
Geiger, P. T., Khansur, N. H., Riess, K., Martin, A., Hinterstein, M. & Webber, K. G. (2018). *J. Appl. Phys.* **123**, 075104.
Gozzo, F., De Caro, L., Giannini, C., Guagliardi, A., Schmitt, B. & Prodi, A. (2006). *J. Appl. Crystallogr.* **39**, 347–357.
Gozzo, F., Schmitt, B., Bortolamedi, T., Giannini, C., Guagliardi, A., Lange, M., Meister, D., Maden, D., Willmott, P. & Patterson, B. D. (2004). *J. Alloys Compd.* **362**, 206–217.
Hastings, J. B., Thomlinson, W. & Cox, D. E. (1984). *J. Appl. Crystallogr.* **17**, 85–95.
Herklotz, M., Scheiba, F., Hinterstein, M., Nikolowski, K., Knapp, M., Dippel, A.-C., Giebeler, L., Eckert, J. & Ehrenberg, H. (2013). *J. Appl. Crystallogr.* **46**, 1117–1127.
Hinterstein, M., Lee, K.-Y., Esslinger, S., Glaum, J., Studer, A. J., Hoffman, M. & Hoffmann, M. J. (2019). *Phys. Rev. B*. **99**, 174107.
Hinterstein, M., Mgbemere, H. E., Hoelzel, M., Rheinheimer, W., Adabifiroozjaei, E., Koshy, P., Sorrell, C. C. & Hoffman, M. (2018). *J. Appl. Crystallogr.* **51**, 670–678.
Hodeau, J.-L., Bordet, P., Anne, M., Prat, A., Fitch, A. N., Dooryhee, E., Vaughan, G. & Freund, A. K. (1998). *Crystal and Multilayer Optics*, Vol. *3448*, edited by A.T. Macrander, A.K. Freund, T. Ishikawa & D.M. Mills, p. 353. SPIE.
Horst, A., Berghaeuser, A., Hinterstein, M. & Knapp, M. (2013). Multi-crystal analyzer detector system for use as detector in synchrotron for analysis of powders, has beam limiting device, which divides overall beam into individual beams and absorbs scattered radiation.
Jin, Y. M., Wang, Y. U. & Khachaturyan, A. G. (2003). *Phys. Rev. Lett.* **91**, 1–4.
Keeble, D. S., Benabdallah, F., Thomas, P. A., Maglione, M. & Kreisel, J. (2013). *Appl. Phys. Lett.* **102**, 092903.
Knapp, M., Baehtz, C., Ehrenberg, H. & Fuess, H. (2004). *J. Synchrotron Radiat.* **11**, 328–334.
Lausi, A., Polentarutti, M., Onesti, S., Plaisier, J. R., Busetto, E., Bais, G., Barba, L., Cassetta, A., Campi, G., Lamba, D., Pifferi, A., Mande, S. C., Sarma, D. D., Sharma, S. M. & Paolucci, G. (2015). *Eur. Phys. J. Plus*. **130**, 43.
Lee, K.-Y., Shi, X., Kumar, N., Hoffman, M., Etter, M., Checchia, S., Silva, L. L. da, Seifert, D. & Hinterstein, M. (2020). *Materials (Basel).* submitted.
Lee, K.-Y., Shi, X., Kumar, N., Hoffman, M., Etter, M., Checchia, S., Winter, J., Lemos da Silva, L., Seifert, D. & Hinterstein, M. (2020). *Materials (Basel).* **13**, 1054.
Lee, K.-Y., Shi, X., Kumar, N., Hoffman, M., Etter, M., Winter, J., Lemos da Silva, L., Seifert, D. & Hinterstein, M. (2020). *Appl. Phys. Lett.* **116**, 182902.
Lee, P. L., Shu, D., Ramanathan, M., Preissner, C., Wang, J., Beno, M. A., Von Dreele, R. B., Ribaud, L., Kurtz, C., Antao, S. M., Jiao, X. & Toby, B. H. (2008). *J. Synchrotron Radiat.* **15**, 427–432.
Liu, L., Knapp, M., Ehrenberg, H., Fang, L., Fan, H., Schmitt, L. A., Fuess, H., Hoelzel, M., Dammak, H., Thi, M. P. & Hinterstein, M. (2017). *J. Eur. Ceram. Soc.* **37**, 1387–1399.
Liu, L., Knapp, M., Ehrenberg, H., Fang, L., Schmitt, L. A., Fuess, H., Hoelzel, M. & Hinterstein, M. (2016). *J. Appl. Crystallogr.* **49**, 574–584.
Liu, L., Knapp, M., Schmitt, L. A., Ehrenberg, H., Fang, L., Fuess, H., Hoelzel, M. & Hinterstein, M. (2016). *Europhys. Lett.* **114**, 47011.
Liu, Q., Zhang, Y., Gao, J., Zhou, Z., Yang, D., Lee, K.-Y., Studer, A., Hinterstein, M., Wang, K., Zhang, X., Li, L. & Li, J.-F. (2020). *Natl. Sci. Rev.* **7**, 355–365.
Masson, O., Dooryhée, E., Cheary, R. W. & Fitch, A. N. (2001). *Mater. Sci. Forum*. **378**–**381**, 300–307.
Mgbemere, H. E., Schneider, G. A., Schmitt, L. A. & Hinterstein, M. (2017). *J. Ceram. Sci. Technol.* **8**, 45–52.
Parrish, W., Hart, M., Erickson, C. G., Masciocchi, N. & Huang, T. C. (1985). *Adv. X-Ray Anal.* **29**, 243–250.
Parrish, W., Hart, M. & Huang, T. C. (1986). *J. Appl. Crystallogr.* **19**, 92–100.

Patterson, B. D., Brönnimann, C., Maden, D., Gozzo, F., Groso, A., Schmitt, B., Stampanoni, M. & Willmott, P. R. (2005). *Nuclear Instruments and Methods in Physics Research, Section B: Beam Interactions with Materials and Atoms*, Vol. *238*, pp. 224–228.
Pawley, G. S. (1981). *J. Appl. Crystallogr.* **14**, 357–361.
Peral, I., McKinlay, J., Knapp, M. & Ferrer, S. (2011). *J. Synchrotron Radiat.* **18**, 842–850.
Ren, Y. & Zuo, X. (2018). *Small Methods*. **2**, 1800064.
Riess, K., Geiger, P. T., Khansur, N. H., Steiner, S., Frömling, T., Hinterstein, M. & Webber, K. G. (2019). *Materialia*. **6**, 100297.
Rodríguez-Carvajal, J. (1993). *Phys. B Condens. Matter*. **192**, 55–69.
Rouquette, J., Hinterstein, M., Haines, J., Papet, P., Knapp, M., Glaum, J. & Fuess, H. (2012). *Acta Cryst. A*, Vol. *68*, p. s94.
Sanlialp, M., Shvartsman, V. V., Acosta, M., Dkhil, B. & Lupascu, D. C. (2015). *Appl. Phys. Lett.* **106**, 062901.
Schader, F. H., Wang, Z., Hinterstein, M., Daniels, J. E. & Webber, K. G. (2016). *Phys. Rev. B*. **93**, 134111.
Schmitt, L. A., Kungl, H., Hinterstein, M., Riekehr, L., Kleebe, H.-J., Hoffmann, M. J., Eichel, R.-A. & Fuess, H. (2013). *2013 Joint IEEE International Symposium on Applications of Ferroelectric and Workshop on Piezoresponse Force Microscopy (ISAF/PFM)*, Vol. pp. 149–151. IEEE.
Shi, X., Ghose, S. & Dooryhee, E. (2013). *J. Synchrotron Radiat.* **20**, 234–242.
Staub, U., Shi, M., O'conner, A. G., Kramer, M. J. & Abrikosov, A. A. (2001). *Phys. Rev. B - Condens. Matter Mater. Phys.* **63**, 1–7.
Staub, U., Soderholm, L. & Wasserman, S. (2000). *Phys. Rev. B - Condens. Matter Mater. Phys.* **61**, 1548–1554.
Supplemental Information (2020). *J. Synchrotron Radiat.*
Sutter, J. P., Chater, P. A., Hillman, M. R., Keeble, D. S., Tucker, M. G. & Wilhelm, H. (2016). *AIP Conference Proceedings*, Vol. *1741*, p. 040005.
Thompson, P., Cox, D. E. & Hastings, J. B. (1987). *J. Appl. Crystallogr.* **20**, 79–83.
Thompson, S. P., Parker, J. E., Potter, J., Hill, T. P., Birt, A., Cobb, T. M., Yuan, F. & Tang, C. C. (2009). *Rev. Sci. Instrum.* **80**, 075107.
Toraya, H. (2009). *J. Appl. Crystallogr.* **42**, 485–489.
Toraya, H., Hibino, H. & Ohsumi, K. (1996). *J. Synchrotron Radiat.* **3**, 75–83.
Vaughan, G. B. M., Baker, R., Barret, R., Bonnefoy, J., Buslaps, T., Checchia, S., Duran, D., Fihman, F., Got, P., Kieffer, J., Kimber, S. A. J., Martel, K., Morawe, C., Mottin, D., Papillon, E., Petitdemange, S., Vamvakeros, A., Vieux, J.-P. & Di Michiel, M. (2020). *J. Synchrotron Radiat.* **27**, 515–528.
Wallwork, K. S., Kennedy, B. J. & Wang, D. (2007). *AIP Conference Proceedings*, Vol. *879*, pp. 879–882. AIP.
Wang, J., Toby, B. H., Lee, P. L., Ribaud, L., Antao, S. M., Kurtz, C., Ramanathan, M., Von Dreele, R. B. & Beno, M. A. (2008). *Rev. Sci. Instrum.* **79**, 085105.
Wang, Z., Webber, K. G., Hudspeth, J. M., Hinterstein, M. & Daniels, J. E. (2014). *Appl. Phys. Lett.* **105**, 161903.
Whittle, T. A., Brant, W. R., Hester, J. R., Gu, Q. & Schmid, S. (2017). *Dalt. Trans.* 7253–7260.
Whittle, T. A. & Schmid, S. (2014). *Powder Diffr.* **29**, S15–S18.
Whittle, T. A., Schmid, S. & Howard, C. J. (2015). *Acta Crystallogr. Sect. B Struct. Sci. Cryst. Eng. Mater.* **71**, 342–348.
Whittle, T. A., Schmid, S. & Howard, C. J. (2018). *Acta Crystallogr. Sect. B Struct. Sci. Cryst. Eng. Mater.* **74**, 742–744.
Whittle, T., Lu, T., Blanchard, P., Hester, J., Gu, Q., Liu, Y. & Schmid, S. (2020). *CrystEngComm*.
Yang, T. Y., Wen, W., Yin, G. Z., Li, X. L., Gao, M., Gu, Y. L., Li, L., Liu, Y., Lin, H., Zhang, X. M., Zhao, B., Liu, T. K., Yang, Y. G., Li, Z., Zhou, X. T. & Gao, X. Y. (2015). *Nucl. Sci. Tech.* **26**, 1–5.
Yavuz, M., Knapp, M., Indris, S., Hinterstein, M., Donner, W. & Ehrenberg, H. (2015). *J. Appl. Crystallogr.* **48**, 1699–1705.
Zhukov, S., Acosta, M., Genenko, Y. A. & von Seggern, H. (2015). *J. Appl. Phys.* **118**, 134104.
